# Heterologous Expression and Characterization of a pH-Stable Chitinase from *Micromonospora aurantiaca* with a Potential Application in Chitin Degradation

**DOI:** 10.3390/md22060287

**Published:** 2024-06-20

**Authors:** Han-Zhong Guo, Dou Wang, Hui-Ting Yang, Yu-Le Wu, Yong-Cheng Li, Guang-Hua Xia, Xue-Ying Zhang

**Affiliations:** 1 Hainan Engineering Research Center of Aquatic Resources Efficient Utilization in South China Sea, Key Laboratory of Food Nutrition and Functional Food of Hainan Province, Key Laboratory of Seafood Processing of Haikou, National R&D Branch Center for Prawn Processing Technology (Haikou), School of Food Science and Engineering, Hainan University, Haikou 570228, China; guohanzhong0622@163.com (H.-Z.G.); wangdouu@126.com (D.W.); yanghuiting000701@163.com (H.-T.Y.); 17370514528@163.com (Y.-L.W.); lyc2360@sina.com (Y.-C.L.); xiaguanghua2011@126.com (G.-H.X.); 2 Collaborative Innovation Center of Provincial and Ministerial Co-Construction for Marine Food Deep Processing, Dalian Polytechnic University, Dalian 116034, China

**Keywords:** *Micromonospora aurantiaca*, chitin, chitinase, characterization, *N*-acetyl chitooligosaccharides

## Abstract

To promote the bioconversion of marine chitin waste into value-added products, we expressed a novel pH-stable *Micromonospora aurantiaca*-derived chitinase, *Ma*Chi1, in *Escherichia coli* and subsequently purified, characterized, and evaluated it for its chitin-converting capacity. Our results indicated that *Ma*Chi1 is of the glycoside hydrolase (GH) family 18 with a molecular weight of approximately 57 kDa, consisting of a GH18 catalytic domain and a cellulose-binding domain. We recorded its optimal activity at pH 5.0 and 55 °C. It exhibited excellent stability in a wide pH range of 3.0–10.0. Mg^2+^ (5 mM), and dithiothreitol (10 mM) significantly promoted *Ma*Chi1 activity. *Ma*Chi1 exhibited broad substrate specificity and hydrolyzed chitin, chitosan, cellulose, soluble starch, and *N*-acetyl chitooligosaccharides with polymerization degrees ranging from three to six. Moreover, *Ma*Chi1 exhibited an endo-type cleavage pattern, and it could efficiently convert colloidal chitin into *N*-acetyl-D-glucosamine (GlcNAc) and (GlcNAc)_2_ with yields of 227.2 and 505.9 mg/g chitin, respectively. Its high chitin-degrading capacity and exceptional pH tolerance makes it a promising tool with potential applications in chitin waste treatment and bioactive oligosaccharide production.

## 1. Introduction

The fishing industry markedly contributes to the global food supply. However, the low-commercial-value marine biomass produced also has a negative effect on the environment and ecosystem when not utilized effectively. Every year, approximately 9 × 10^9^ tons of crustaceans including crabs, shrimps, and lobsters are generated, with roughly 60% being disposed of as solid waste due to their inedibility [1,2]. This crustacean waste, containing 15–40% chitin [3], presents a cheap and renewable resource. Therefore, converting traditionally discarded crustacean waste into value-added bio-products using an effective and eco-friendly approach is critical for developing sustainable seafood systems and enhancing the global carbon and nitrogen cycle.

Chitin, the second most prevalent natural polysaccharide following cellulose, is synthesized from *N*-acetyl-D-glucosamine (GlcNAc) through *β*-1,4 glycosidic linkages [4]. Its hydrolysis products can contribute significantly to the shift toward a bioeconomy. For example, the chitin monomer (GlcNAc) is a promising small-molecule compound with various biological activities, such as anti-tumor and anti-oxidant properties, and it is clinically useful in treating osteoarthritis and rheumatoid arthritis [5,6]. GlcNAc also serves as fermentation sugar for the microbial production of biofuels [7]. Dimeric *N*-acetyl chitobiose (GlcNAc)_2_, a highly valuable *N*-acetyl chitooligosaccharide (NCOS) with anti-oxidant, antiviral, metabolic regulatory, lipid-lowering, chelating, and hypoglycemic effects, can also be used to produce bioactive oligomers [8,9,10]. As potential functional materials in the agricultural, food, pharmaceutical, cosmetic, and other industries, the growing interest in these products has led to an increased desire for GlcNAc and (GlcNAc)_2_ [11,12]. However, chitin has a crystalline structure with numerous intra- and inter-molecular hydrogen bonds, making it insoluble in aqueous media. Therefore, although chitin resources are abundant in nature, the amount of chitin utilized by humans is very small, causing significant resource wastage and environmental harm.

Currently, GlcNAc and (GlcNAc)_2_ are predominantly obtained from chitin through acid hydrolysis at elevated temperatures. However, this approach has several disadvantages, including low yield, product instability, uncontrollable processing, and environmental risks [13]. In contrast, enzymatic chitin hydrolysis under mild conditions is a sustainable and eco-friendly alternative method for GlcNAc and (GlcNAc)_2_ production.

Chitinases are commonly used glycoside hydrolases (GHs) in the enzymatic degradation of chitin that specifically cleave the *β*-1, 4 glycosidic linkages in a polysaccharide chain [14]. Based on their mechanism of action, chitinases are categorized as *endo*- or *exo*-chitinases. The former randomly cleaves glycosidic bonds from the interior of chitin to generate GlcNAc or soluble oligomers, whereas the latter hydrolyzes glycosidic bonds from chitin’s reducing or non-reducing ends to release (GlcNAc)_2_ [15]. Furthermore, depending on the similarities of their amino acid sequences, chitinases are mainly divided into GH18 and 19 families, along with a small number of chitinases that are classified into the GH23 or GH48 families [16]. Chitinases are widely found in bacteria, fungi, insects, and mammals [17]. Many marine and soil bacteria can utilize chitin as sources of carbon and nitrogen due to their capacity to secrete adequate chitinases [18], and these bacterial chitinases have attracted more interest than others. Several bacterial chitinases have been identified for their degradation activities toward chitinous substrates, such as the recombinant chitinase Chisb from *Bacillus* sp. DAU101, which hydrolyzed colloidal chitin into a mixture of (GlcNAc)_1-5_ [19]; wild-type chitinase ChiTg from *Trichoderma gamsii* R1, which converted colloidal chitin into (GlcNAc)_1-3_ [2]; and the recombinant chitinase CcCti1 from *Corallococcus* sp. EGB, which could degrade colloidal chitin into (GlcNAc)_6_ as its major product [20]. However, most chitinases are mesophilic or neutral, making them unsuitable for the harsh conditions encountered during industrial production. For wild-type enzymes, the complicated and high-cost purification procedures also hinder mass production and application. Therefore, on the one hand, novel chitinases with atypical features (e.g., wide pH tolerance and good thermal stability) should be explored; on the other hand, a simple and affordable process should be applied for chitinase purification, being important for industrial applications.

*Actinomycetales* are highly effective at breaking down chitin, cellulose, lignin, and other polysaccharides [21]. *Micromonospora* sp., a Gram-positive soil actinobacterium, is widely found in nature and its chitinolytic activity has previously been reported upon [22,23,24]. However, only a few chitinolytic enzymes from *Micromonospora* sp. have been purified and identified [25,26]. In this study, we aimed to identify *Ma*Chi1, an *M. aurantiaca*-derived innovative GH18 chitinase with a broad range of pH tolerance by aligning sequences, analyzing its structure, and expressing it in *Escherichia coli*. Furthermore, we sought to purify this chitinase, using simple affinity chromatography, and characterize its enzymatic properties, substrate specificity, and degradation mechanism. Finally, we evaluated the potential applications of this enzyme in chitin conversion.

## 2. Results and Discussion

### 2.1. Sequence Analysis of MaChi1

**Figure 1:**
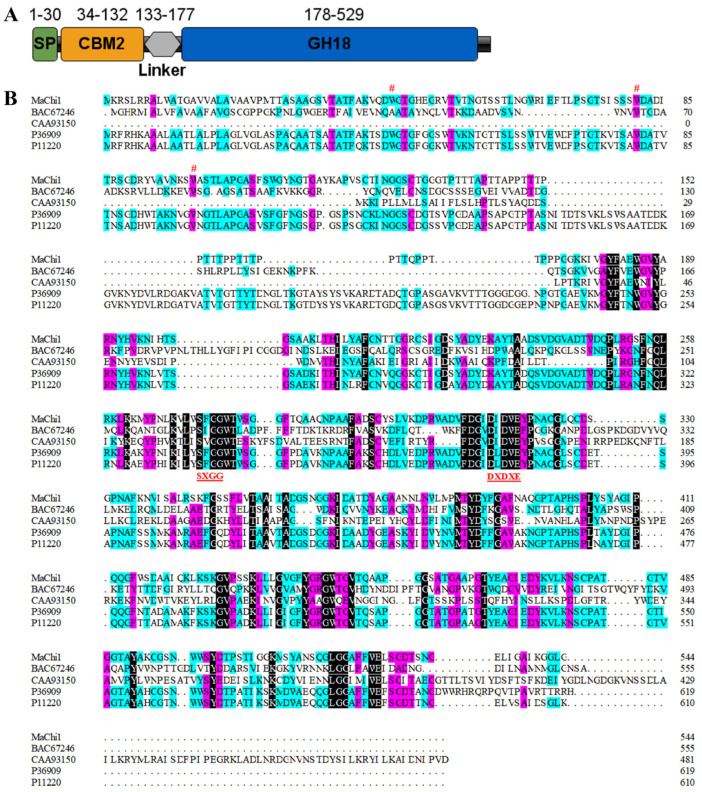
Sequence analysis of *Ma*Chi1. (**A**) The domain structure of *Ma*Chi1. This image was generated using IBS Illustrator [35]. (**B**) Multi-sequence alignment of *Ma*Chi1 from *M. aurantiaca* with other GH18 chitinases from *Bombyx mori* (accession number: BAC67246), *Acetiyibrio thermocellus* ATCC 27,405 (CAA93150), *Streptomyces lividans* (P36909), and *Streptomyces plicatus* (P11220). Conserved amino acid residues (Trp^44^, Trp^81^, and Trp^99^) are marked with a (#) sign. Conserved sequence motifs essential for catalysis (^273^SXGG^276^ and ^314^DXDXE^318^) are highlighted at the bottom.

The full length of the *Ma*Chi1 gene sequence is 1632 bp (Figure S1) (Supplementary Materials), which encodes a protein containing 544 amino acids (Figure S2). The estimated molecular weight (MW) and predicted isoelectric point (pI) of *Ma*Chi1 protein are 57 kDa and 8.7, respectively. Amino acid sequence alignment revealed that *Ma*Chi1 exhibited 73% identity with chitinase C from *Streptomyces lividans* (P36909), 70% identity with chitinase 63 from *Streptomyces plicatus* (P11220), 35% identity with chitinase A1 from *Niallia circulans* (P20533), and 32% identity with chitinase A from *Pseudoalteromonas piscicida* (P32823). *Ma*Chi1 contains multiple structural domain modules, including a signal peptide (SP), a cellulose-binding domain (CBD), a Thr/Pro-rich linker domain, and a GH18 catalytic domain (Figure 1A). The CBD from *Ma*Chi1 belongs to the carbohydrate-binding module family 2 (CBM2), known for its specific binding to cellulose, xylan, and chitin [27,28,29]. Conserved tryptophan residues, which are essential for carbohydrate binding [30,31], are present in the CBD of *Ma*Chi1 (Figure 1B). The catalytic domain of *Ma*Chi1 contains extremely conserved catalytic center motif (DXDXE) and chitin-binding motif (SXGG), typical of GH18 chitinases (Figure 1B).

**Figure 2:**
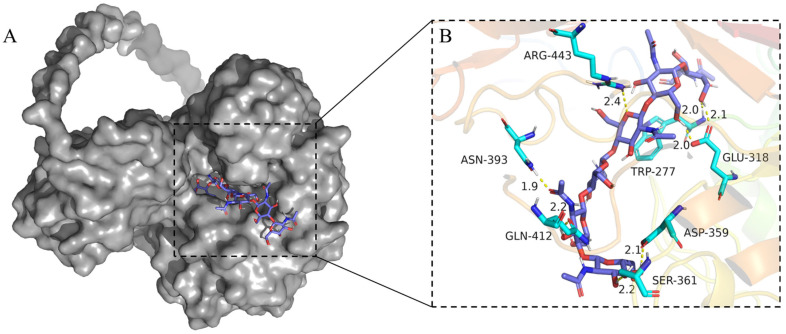
Docking of the (Gl_C_NAc)_6_ molecule in the catalytic site of *Ma*Chi1. (**A**) Docked conformation of (Gl_C_NAc)_6_ (blue sticks) in *Ma*Chi1’s catalytic groove (gray surface). (**B**) Detailed view of (Gl_C_NAc)_6_ and its possible interactions with the surrounding residues of *Ma*Chi1. Hydrogen bonds are represented as yellow dotted lines. Distances (in Å) are represented as black numbers.

The three-dimensional (3D) model of *Ma*Chi1, excluding the signal peptide, was built using AlphaFold2. Upon per-residue confidence metric analysis, the model showed a generally accurate backbone prediction [32,33]. Apart from the linker domain, the model of the CBD and GH18 catalytic domains was established confidently, with a predicted local distance difference test (pLDDT) value higher than 70 (Figure S3). In this model, the catalytic domain of *Ma*Chi1 was composed of a (*β*/*α*)_8_-barrel fold, consistent with the structural characteristics commonly found in GH18 chitinases. The CBD of *Ma*Chi1 was modeled as a compact structure comprising an anti-parallel *β*-sheet containing seven *β*-strands linked by loops (Figure S4). To elucidate how the substrate interacts with its surrounding amino acid residues, the molecular docking of (GlcNAc)_6_ to the active site of *Ma*Chi1 was conducted. The modeled protein–ligand complex revealed a network of hydrogen bonds and CH–π interactions, with hydrogen bonds serving as the major forces to stabilize the conformation of the *Ma*Chi1–(GlcNAc)_6_ complex. In addition to the putative catalytic Glu^318^, six other residues (Trp^277^, Arg^443^, Asn^393^, Gln^412^, Ser^361^, and Asp^359^) interacted with the monosaccharide residues through hydrogen bonds. Two residues, Phe^392^ and Trp^277^, generated evident CH–π interactions with the substrate (Figure 2 and Figure S5). CH–π interactions mainly involve the aromatic residues and play significant roles in promoting tighter binding and enhanced processivity [34]. However, the amino acid residues involved in CH–π interactions are less conserved, whereas those forming hydrogen bonds with substrate are highly conserved. This observation was consistent with the finding that hydrogen bonds constitute the major stabilizing force in GH18 chitinases.

### 2.2. Expression and Purification of MaChi1

In this study, *Ma*Chi1 lacking the SP was successfully expressed in *E. coli* BL21(DE3) cells as an active protein. It was purified to electrophoresis purity, with purification multiple and recovery rate being 27.5 and 84.6%, respectively (Table S1). Sodium dodecyl sulfate (SDS)-polyacrylamide gel electrophoresis (PAGE) analysis estimated the MW to be 57 kDa (Figure S6), which matches the estimation based on the amino acid sequences.

### 2.3. Enzymatic Characterization of MaChi1

**Figure 3:**
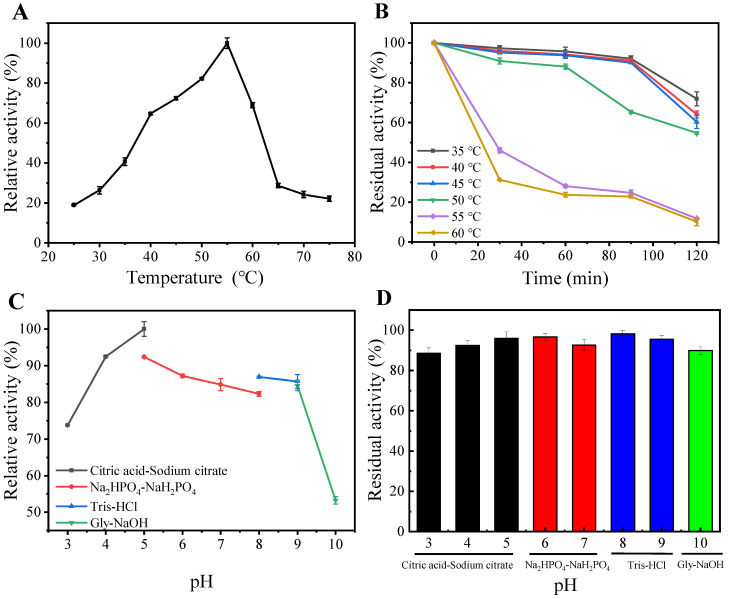
(**A**) Optimal temperature, (**B**) thermostability, (**C**) optimal pH, and (**D**) pH stability of *Ma*Chi1.

The impact of temperature on the performance of purified *Ma*Chi1 was evaluated using temperatures between 25 and 75 °C and colloid chitin as the substrate. *Ma*Chi1 displayed a maximum activity at 55 °C (Figure 3A), which is similar to the chitinases found in *Microbispora* sp. V2 [36], *Streptomyces sampsonii* XY2-7 [37], and *Streptomyces violascens* Nag2557 [38]. The enzymic activity decreased significantly when the reaction temperature was above 55 °C; the relative activity at 65 °C was only 28.7% (Figure 3A). Regarding thermostability, *Ma*Chi1 retained over 88.2% of its original activity after being exposed to temperatures between 35 and 50 °C for 1 h and retained over 54.7% of its original activity after 2 h (Figure 3B). Nevertheless, the poor thermal stability of chitinase was observed at its optimal temperature, with 71.9% of activity lost after incubation for 1 h (Figure 3B).

The relative activity of *Ma*Chi1 exceeded 82.3% within the pH range of 4.0–9.0, and the maximum enzyme activity was recorded at pH 5.0 (Figure 3C), suggesting that *Ma*Chi1 is an acidic chitinase. Similar optimal pH values were observed for the chitinases from *B. subtilis* and *Streptomyces albolongus* ATCC 27414; however, they exhibited much narrower optimal pH ranges than *Ma*Chi1 [39,40]. Additionally, other neutral and alkaline chitinases with optimal pH values between 7.0 and 10.0 have been reported [41,42,43]. The residual activity of *Ma*Chi1 was retained above 88.6% after incubation for 1 h at 4 °C throughout a wide pH range of 3.0 to 10.0 (Figure 3D), indicating its remarkable pH stability. The pH range for *Ma*Chi1’s enzymic activity is broader than that for some other chitinases (Table 1). For instance, *Sa*HEX from *Streptomyces alfalfa* was stable within a narrow pH range of 4.5–8.5 [44], and *Ss*Chi18C from *Streptomyces* sp. F-3 maintained above 80% of its original activity between pH 3.0 and 8.0 [45]. Furthermore, Chi1602 from *Microbulbifer* sp. BN3 showed more than 80% residual activity within the pH range of 5.0–9.0 [46]. Considering the good thermal stability and broad pH profile of *Ma*Chi1, it has the potential for wider industrial applications.

The activity of *Ma*Chi1 was suppressed by K^+^, Ag^+^, Co^2+^, Zn^2+^, Cu^2+^, Fe^2+^, and Fe^3+^. Except for Ag^+^, the inhibition intensified with increasing concentration of the aforementioned metal ions. Conversely, *Ma*Chi1 activity increased with increasing concentrations of Mg^2+^. Moreover, Ca^2+^ and Ba^2+^ slightly increased *Ma*Chi1 activity at low (1 mM) concentrations but inhibited chitinase activity at high (5 mM) concentrations (Table S2). The influence of chemical agents on *Ma*Ch1’s activity is summarized in Table S3. At the tested concentrations, urea reduced chitinase activity to 85.8% of its original activity. Regarding surfactants, chitinase activity was affected to a low degree by Tween-20, -40, and -80 and Triton X 100, whereas SDS and Tween-60 caused a 25.1–38.1% reduction in the original activity. Reducing agents such as *β*-mercaptoethanol and dithiothreitol (DTT) increased chitinase activity to 104.1% and 144.8% of its original level, respectively. The chelator ethylene diamine tetraacetic acid (EDTA) had an insignificant influence on enzyme activity, indicating that *Ma*Chi1 is not reliant on metal ions for its function.

**Table 1:** Comparison of enzymatic properties of *Ma*Chi1 with chitinases from other organisms.

Organism	Chitinase	MW (kDa)	Optimal Temperature (°C)	Optimal pH	pH Stability	Ref
*Streptomyces alfalfae*	*Sa*HEX	60	60	5.5; relative activity was above 80% at 5.0–6.0	pH range of 4.5–8.5, incubation for 1 h, residual activity was above 80%	[44]
*Streptomyces* sp. F-3	*Ss*Chi18C	–	60	5.0; relative activity was above 80% at 3.0–7.0	pH range of 3.0–8.0, incubation for 0.5 h, residual activity was above 80%	[45]
*Microbulbifer* sp. BN3	Chi1602	60	60	9.0; relative activity was above 80% at 4.0–9.0	pH range of 5.0–9.0, incubation for 1 h, residual activity was above 80%	[46]
*Paenibacillus* sp.	A1	30	50	4.5; relative activity was above 90% at 4.0–5.0	pH range of 4.5–5.5, incubation for 1 h, residual activity was above 80%	[47]
*Thermophilic* sp.	Chi304	70.95	80	9.0; relative activity was above 80% at 8.0–10.0	pH range of 6.0–10.0, incubation for 1 h, residual activity was above 60%	[48]
*Trichoderma gamsii* R1	ChiTg	42	40	5.0; relative activity was above 70% at 4.0–6.0	pH range of 5.0–8.0, incubation for 0.5 h, residual activity was above 80%	[2]
*Paenibacillus* sp.	Y412MC10	52	60	5.5; relative activity was above 70% at 4.5–6.5	pH range of 4.5–6.5, incubation for 1 h, residual activity was above 90%	[49]
*Chitinilyticum* sp. C8	ChiC8–1	100	50	6.0; relative activity was above 70% at 4.0–9.0	pH range of 5.0–8.0, incubation for 1 h, residual activity was above 90%	[50]
*Aeromonas media* CZW001	*Am*Chi	40	55	8.0; relative activity was above 75% at 6.0–9.0	pH range of 4.0–9.0, incubation for 2 h, residual activity was above 70%	[51]
*Trichoderma harzianum* GIM 3.442	Chit46	46	45	6.0; relative activity was above 80% at 6.0–7.0	pH range of 5.0–9.0, incubation for 1 h, residual activity was above 80%	[52]
*M*. *aurantiaca*	*Ma*Chi1	57	55	5.0; relative activity was above 82% at 4.0–9.0	pH range of 3.0–10.0, incubation for 1 h, residual activity was above 88.6%	This study

### 2.4. Substrate Spectrum and Kinetic Parameters of MaChi1

Table 2 shows that *Ma*Chi1 exhibited a higher activity for colloidal chitin (CC) (1.78 U/mg) and *β*-chitin (1.44 U/mg) than for α-chitin (0.50 U/mg) and shrimp shell powder (0.69 U/mg). The hydrolysis capacity of *Ma*Chi1 toward (GlcNAc)_3-6_ decreased from 3.11 U/mg to 1.15 U/mg with the decreasing degrees of polymerization, and it was found to be inactive toward (GlcNAc)_2_. These results suggest that *Ma*Chi1 has a preference for polysaccharides with a relatively low crystallinity or loosely ordered structures as well as soluble oligomers, which it requires for eliciting further hydrolysis. Furthermore, *Ma*Chi1 exhibited activity against chitosan with a deacetylation degree (DD) of 50–95%, and its hydrolytic activity decreased with an increase in DD. This phenomenon is similar to that of chitinases chip1 and chip2 from *Paenibacillus pasadenensis* CS0611 [53]. Weak activities against starch (0.34 U/mg) and cellulose (0.34 U/mg) were also discovered, which was consistent with the previous studies that reported the high specificity of chitinases for cleaving glycosidic bonds between GlcNAc–GlcNAc, GlcNAc–GlcN, or GlcN–GlcNAc [54,55]. In some cases, chitinases show strict substrate specificity, showing no activity against non-chitinous substrates like starch, cellulose, or carboxymethyl cellulose [52,56,57].

As shown in Table 3, the kinetic parameters *V_max_*, *K_m_*, *K_cat_*, and *K_cat_*/*K_m_* of *Ma*Chi1 for colloidal chitin were determined to be 2.33 μmol·min^−1^·mg^−1^, 4.60 mg/mL, 0.43 s^−1^, and 0.09 mg^−1^·mL·s^−1^, respectively. *Ma*Chi1 exhibited stronger substrate affinity for CC than *Sa*ChiB from *Streptomyces alfalfa* (9.68 mg/mL) [42], *Pb*Chi70 from *Paenibacillus barengoltzii* (7.91 mg/mL) [58], and k10 from *Penicillium oxalicum* k10 (12.56 mg/mL) [59], as suggested by its lower *K*_m_ value.

### 2.5. Affinity of MaChi1 for Polysaccharides

**Figure 4:**
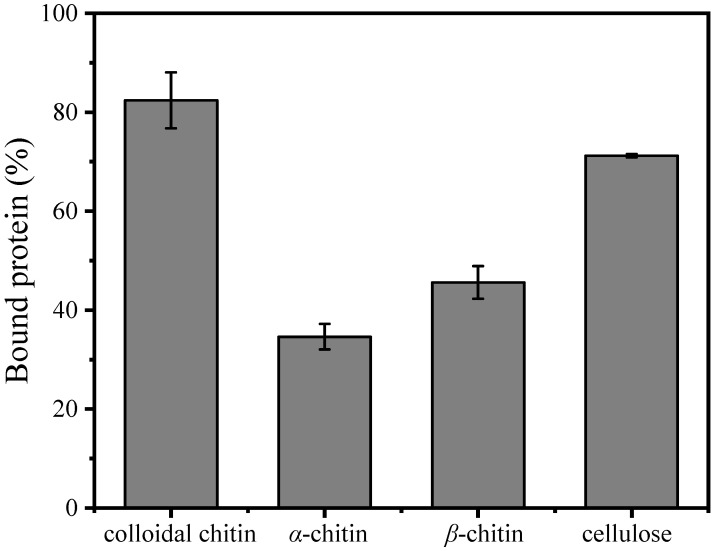
Binding ability of purified *Ma*Chi1 to the substrate.

The substrate-binding ability of *Ma*Chi1 toward insoluble substrates is shown in Figure 4. *Ma*Chi1 showed a strong affinity toward cellulose (71.22%), likely attributed to the presence of the CBM2 domain (Figure 1A), which is primarily associated with cellulose binding. Meanwhile, a moderate affinity toward *α*-chitin (34.62%) and *β*-chitin (45.61%) was observed, suggesting that *Ma*Chi1’s affinity was not specific to cellulose. Moreover, of the evaluated insoluble polysaccharides, the binding activity toward CC (82.52%) was the highest. According to the Carbohydrate-Active Enzymes (CAZy) database, CBM2 is mainly found in carbohydrate hydrolases like xylanases or cellulases that can hydrolyze *β*-(1, 4)-glucans; it is uncommon among chitinases. Similarly, *Bth*Chi74 from *Bacillus thuringiensis*, which contains the CBM2 domain, showed binding affinity for both cellulose and chitinous substrates [29]. Compared with *α*-chitin and *β*-chitin, the less smooth and more porous structure of CC may increase protein absorption and make hydrolysis easier, as reported by Zhang et al. [60]. Furthermore, the strong affinity (71.22%) but low specific enzyme activity (0.34 U/mg, Table 2) of chitinase toward cellulose indicated that the more-affordable cellulose may be a promising adsorbent for *Ma*Chi1 purification.

### 2.6. Hydrolytic Property of MaChi1

**Figure 5:**
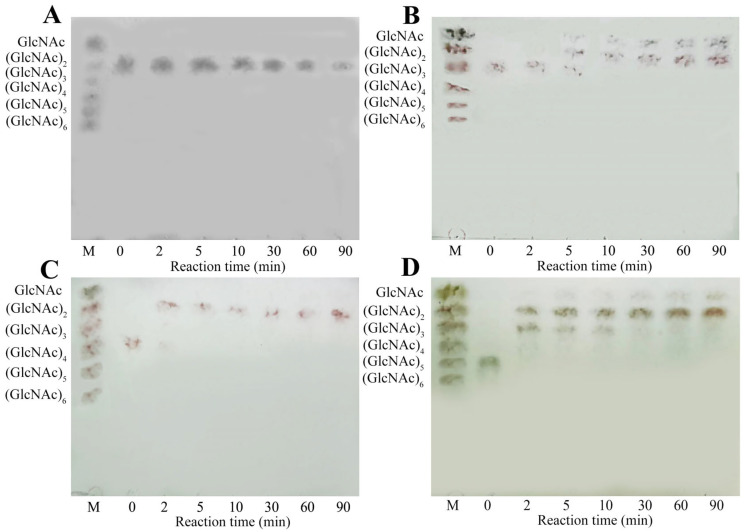
TLC analysis of hydrolysis products from (**A**) (GlcNAc)_2_, (**B**) (GlcNAc)_3_, (**C**) (GlcNAc)_4_, and (**D**) (GlcNAc)_5_ with *Ma*Chi1.

The hydrolytic properties of *Ma*Chi1 toward (GlcNAc)_2–5_ were determined using thin-layer chromatography (TLC). Consistent with the substrate specificity findings (Table 2), *Ma*chi1 was incapable of hydrolyzing (GlcNAc)_2_ (Figure 5A). When (GlcNAc)_3_ was employed as the substrate, (GlcNAc)_2_ and GlcNAc were obtained as the final products (Figure 5B). During the reaction period, the hydrolysis of (GlcNAc)_4_ resulted in the formation of (GlcNAc)_2_ only, with no detection of GlcNAc or (GlcNAc)_3_ (Figure 5C), suggesting that *Ma*Chi1 has a binding preference for the −2 to +2 site. For (GlcNAc)_5_, the hydrolysis products observed during the first 10 min were predominantly (GlcNAc)_3_ and (GlcNAc)_2_. Only minimal amounts of (GlcNAc)_4_ and GlcNAc were detected via TLC imaging. Subsequently, the generated (GlcNAc)_3_ was progressively broken down into (GlcNAc)_2_ and GlcNAc as the reaction time increased from 30 to 90 min (Figure 5D). Given that an odd number of sugars are detected at the beginning of the reaction, *Ma*Chi1 may be a non-processive *endo*-type chitinase that has a strong capacity to produce (GlcNAc)_2_, similar to other chitinases like *Pb*Chi67 from *Paenicibacillus barengoltzii* CAU904 [61] and ChiA-Hh59 from *Hydrogenophilus hirschii* KB-DZ44 [58].

### 2.7. Hydrolysis of Colloidal Chitin

**Figure 6:**
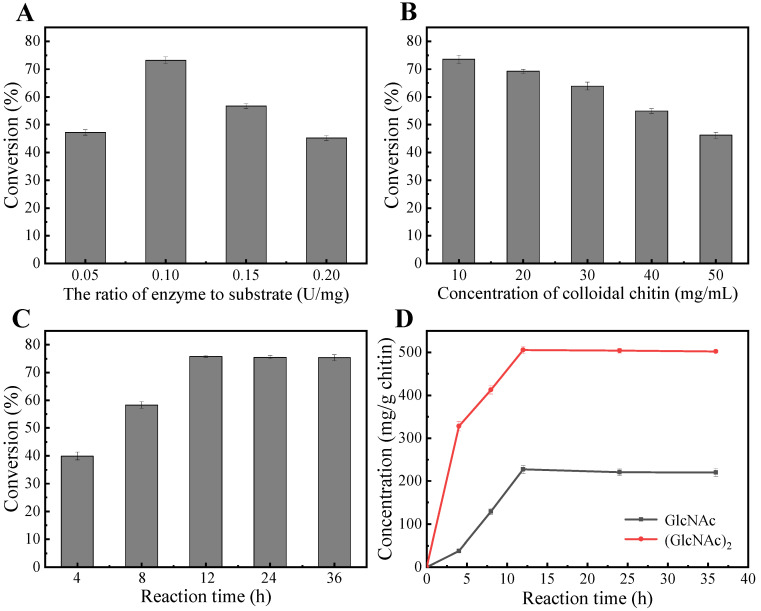
Preparation of *N*-acetyl chitooligosaccharides from colloidal chitin. Optimization of (**A**) the enzyme-to-substrate ratio, (**B**) colloidal chitin concentration, and (**C**,**D**) reaction time.

Based on our findings regarding the enzymatic properties of *Ma*Chi1 (Figure 3), the subsequent reactions for hydrolyzing CC were performed in a citrate buffer with a pH of 5.0 at a temperature of 50 °C. As shown in Figure 6A, with an increased enzyme-to-substrate ratio, the conversion of CC first increased and then decreased. The highest value was observed for 0.1 U/mg. Figure 6B illustrates the influence of substrate concentration on hydrolysis efficiency. The CC conversion rate was 73.6% at 10 mg/mL but fell significantly to 46.2% at 50 mg/mL. Under the optimal reaction conditions, the conversion rate of CC reached its highest value (75.9%) after 12 h (Figure 6C). GlcNAc and (GlcNAc)_2_ produced 227.2 and 505.9 mg/g chitin, respectively (Figure 6D). Recently, the degradation of chitin by enzymatic digestion has attracted increasing attention due to its green properties. For instance, Vaikuntapu et al. [62] used chitinase *Fj*ChiB from *Flavobacterium johnsoniae* UW101 to hydrolyze CC, resulting in yields of 59 mg/g chitin for (GlcNAc)_2_ and 47 mg/g chitin for (GlcNAc)_3_. Gao et al. [40] used the chitinase *Sa*ChiA4 from *Streptomyces albolongus* to break down CC, leading to yields of 8.8 mg/g chitin for GlcNAc and 21.9 mg/g chitin for (GlcNAc)_2_. Fu et al. [55] reported the conversion of CC into GlcNAc, (GlcNAc)_2_, and (GlcNAc)_3_ in the presence of the chitinase *Ea*Chi39 from *Exiguobacterium antarcticum* DW2, and the yields were 330, 490, and 167 mg/g chitin, respectively. Thus, *Ma*Chi1 may serve as an efficient and competitive biocatalyst for degrading chitin biomass to yield GlcNAc and (GlcNAc)_2_.

## 3. Materials and Methods

### 3.1. Materials

Shrimp shells were acquired from a shrimp processing factory in Hainan Province (China) for preparing the shrimp shell powder (particle size 0.25–0.425 mm). *α*-Chitin and GlcNAc were obtained from Aladdin Co. (Shanghai, China). (GlcNAc)_2-6_ were obtained from Qingdio BZ Oligo Biotech Co., Ltd. (Qingdao, China). CC was prepared from *α*-chitin using Joe et al.’s method [63]. *β*-Chitin from squid pens was prepared by the method stated in a study by Yang et al. [64]. Ni-NTA bead columns were purchased from Changzhou Smart-Lifesciences Biotechnology Co., Ltd. (Changzhou, China). All other chemicals used were of reagent or molecular biology grade and obtained from local suppliers.

### 3.2. Strains and Culture Conditions

*M. aurantiaca* was incubated at 30 °C in medium (pH 7.0) consisting of 5 g/L glucose, 10 g/L yeast extract, 1.5 g/L CaCl_2_, 0.3 g/L CC, and 10 g/L NaCl. *Escherichia* DH5α and *E. coli* BL21 (DE3) cells were cultivated at 37 °C in Luria–Bertani (LB) medium (pH 7.2) containing 5 g/L yeast extract, 10 g/L tryptone, and 10 g/L NaCl. When necessary, 100 mg/L of ampicillin (Amp) or 50 mg/L of kanamycin (Kana) was added to the LB medium to maintain plasmid expression.

### 3.3. Cloning, Expression, and Bioinformatic Analysis of the MaChi1 Gene

Genomic DNA from *M. aurantiaca* was used as the PCR template and extracted by using a Rapid Bacterial Genomic DNA Isolation Kit (Sangon, Shanghai, China). Before DNA extraction, the cells were homogenized with a mortar and pestle and liquid nitrogen was added to the mortar to assist in the homogenization process. Two primer sequences, *Ma*Chi1F (5′-GGATCCGCCGGCAGCGTCACCGC-3′) and *Ma*Chi1R (5′-AAGCTTTCAGCCGAGACCGCCCTTGAT-3′), containing *Bam*H I and *Hind* III restriction sites were designed based on the known gene sequence (GenBank Accession No. ADL48027) and synthesized by Sangon Biotech (Shanghai, China). The PCR products and pET28a (+) vector were double-digested with appropriate restriction enzymes and ligated with the T4 DNA enzyme to obtain recombinant expression vectors containing two six-His tags. The recombinant expression vectors were introduced into *E. coli* BL21(DE3) cells, which were then grown in LB medium with 50 mg/L Kana at a 180 rpm shaking speed and 37 °C. When the OD_600_ of the bacterial culture reached 1.2, isopropyl-*β*-D-thiopyranogalactoside (IPTG) at a final concentration of 0.2 mM was added as an inducer. The culture was subsequently maintained at a temperature of 16 °C for another 16 h.

BLAST was employed to analyze *Ma*Chi1’s amino acid sequence. The SignalP 5.0 server was used to analyze the signal peptides. The DNAMAN tool was utilized to perform protein homologous sequence alignment. The NCBI’s CD-Search tool was utilized to analyze the conserved domains. The ExPASy ProtParam program was employed to forecast the MW and pI. AlphaFold2 was used to model the protein molecule’s 3D structure [65]. The molecular docking of *Ma*Chi1 with (GlcNAc)_6_ was investigated using AutoDock Vina 1.1.2 [66]. The ligand (GlcNAc)_6_ was docked to the active sites of *Ma*Chi1 within a 30 × 30 × 30 grid box. The central point of *Ma*Chi1 was set to (−9.92, 1.02, −1.16) on the XYZ coordinates. The 3D structure of *Ma*Chi1 was analyzed using PyMOL 2.5.6. The online websites used for this study are available in Table S4.

### 3.4. Purification of Recombinant MaChi1

Prior to purification, the cells were harvested by centrifugation at 8000 rpm and 4 °C for 10 min and lysed by sonication in an ice water bath to release the soluble proteins. To remove the cell debris, the cell lysates were centrifuged at 8000 rpm and 4 °C for 20 min before being filtered through a 0.22 µm filter. Next, the supernatant was loaded onto a Ni-NTA bead column that was pre-equilibrated with binding buffer (50 mM NaH_2_PO_4_, 300 mM NaCl, and 10 mM imidazole, pH 8). After the supernatant was bound onto the column for 30 min at 4 °C, the hybrid proteins were washed with 5× column volume wash buffer (50 mM NaH_2_PO_4_, 300 mM NaCl, and 50 mM imidazole, pH 8). Finally, the adsorbed target proteins were eluted with elution buffer (50 mM NaH_2_PO_4_, 300 mM NaCl, and 100 mM imidazole, pH 8) and identified using SDS-PAGE.

### 3.5. Chitinase Activity Assay

Chitinase activity was assessed by employing the 3,5-dinitrosalicylic acid (DNS) approach [67]. Briefly, 0.5 mL of 3% (*w*/*v*) CC was mixed with 0.5 mL of purified *Ma*Chi1. The reaction was conducted in 50 mM phosphate buffer (pH 7.0) at 50 °C. After 30 min, the reaction was interrupted by introducing 1 mL of DNS reagent and subsequent heating in a boiling water bath to deactivate the enzyme. Upon cooling, 8 mL of distilled water was supplied to the above solution, followed by centrifugation at 8000 rpm for 10 min. The absorbance values of the supernatant at 540 nm were then determined. One unit (U) of chitinase activity was designated as the quantity of enzyme required to generate 1 μM of GlcNAc per minute. The Bradford method [68] was applied to determine the protein concentration, with bovine serum albumin serving as the reference.

### 3.6. Biochemical Characteristics of MaChi1

The temperature for facilitating the optimal activity of *Ma*Chi1 was measured by employing the standard method at temperatures between 25 and 75 °C. The thermostability of *Ma*Chi1 was assessed through determining the remaining enzyme activities following incubation at temperatures between 35 and 60 °C.

The pH for facilitating the optimal activity of *Ma*Chi1 was measured in buffers at pH values of 3−10 by employing the standard method. The pH stability of *Ma*Chi1 was evaluated by determining the remaining enzyme activities following exposure to the specified buffers (pH 3−10) for 1 h at 4 °C.

The influence of metal ions (K^+^, Ag^+^, Mg^2+^, Fe^2+^, Ca^2+^, Co^2+^, Ba^2+^, Zn^2+^, Cu^2+^, and Fe^3+^), a chelator (EDTA), and denaturants (Tween-20, -40, -60, and -80, urea, *β*-mercaptoethanol, SDS, DTT, and Triton X-100) on *Ma*Chi1 activity was examined through the addition of individual reagents to the reaction system. The enzyme activity was measured by employing the standard method and the relative activity was calculated as the proportion of enzyme activity detected without the addition of any reagent.

### 3.7. Substrate Specificity and Enzyme Kinetics

The hydrolytic activities of *Ma*Chi1 toward shrimp shell powder, CC, *α*-chitin, *β*-chitin, chitosan with DD 55–95%, (GlcNAc)_2–6_, soluble starch, and cellulose were determined at 55 °C in citrate buffer (50 mM, pH 5.0). The substrate’s ultimate concentration in the reaction system was adjusted to 2 mg/mL. As described earlier, a DNS approach was used to assess the extent of substrate hydrolysis.

The apparent kinetic parameters for *Ma*Chi1’s activity toward CC were investigated through determining enzymic activity in a 50 mM citrate buffer at pH 5.0 and 55 °C for 30 min. The final concentrations of CC were 1–12 mg/mL. The *K*_m_, *V*_max_, and *K*_cat_ values were determined using GraphPad Prism 8.0 software.

### 3.8. Substrate-Binding Capacity of MaChi1

A series of substrates, including CC, *α*-chitin, *β*-chitin, and cellulose, was tested to determine the substrate-binding capacity of *Ma*Chi1. The binding reaction mixtures contained 2 mg/mL polysaccharide (as the substrate) and 0.1 mg/mL *Ma*Chi1 in citrate buffer (50 mM, pH 5.0). Following incubation at 25 °C with gentle agitation for 1 h, the enzyme–substrate combinations were removed by centrifugation. The free protein content in the supernatant was determined using the Bradford method [68]. The disparity between the starting and ending protein concentrations in the supernatant was defined as the amount of binding protein. To assess the nonspecific binding of the protein, blanks (buffer + substrate) and controls (buffer + protein) were used simultaneously.

### 3.9. Degradation Mode of MaChi1

To investigate the mechanism of action, the products of (GlcNAc)_2–5_ hydrolyzed by *Ma*Chi1 were analyzed using TLC. Reaction mixtures containing 2 mg/mL of the substrate and 0.5 U/mL of *Ma*Chi1 in citrate buffer (50 mM, pH 5.0) were incubated at 55 °C. Samples were taken from the reaction mixtures periodically between 0 and 90 min and were rapidly boiled to deactivate the enzyme. The products underwent evaluation using TLC following the procedure specified by Guo et al. [69]. A mixture of GlcNAc and (GlcNAc)_2–6_ was used as the standard.

### 3.10. Enzymatic Hydrolysis of Colloidal Chitin

The reaction was performed in 5 mL of citrate buffer (50 mM, pH 5.0) with 50 mg of CC and 5 U of *Ma*Chi1 at 50 °C for 12 h with magnetic stirring (1300 rpm). A single-factor test was carried out to optimize the reaction conditions by varying the enzyme-to-substrate ratio (0.05, 0.10, 0.15, and 0.20 U/mg), concentration of substrate (10, 20, 30, 40, and 50 mg/mL), and reaction time (4, 8, 12, 24, and 36 h). The process ended by boiling the mixture in a water bath for 10 min. After centrifugation, the residual CC was dried to achieve a constant weight at 60 °C. Substrate conversion was defined as the percentage of chitin consumed relative to the initial amount of chitin. The hydrolyzed products in the supernatant were analyzed using high-performance liquid chromatography (HPLC) following Zhang’s method [70].

### 3.11. Statistical Analyses

SPSS 24.0 software was used to analyze the data, and Origin 2021 software was used to draw images. All experiments were conducted in triplicate, and the data are presented as means ± standard deviations.

## 4. Conclusions

In the present study, we characterized a novel GH18 family chitinase (*Ma*Chi1) via its heterologous expression. The sequence alignment result demonstrated the existence of the conserved motif ^314^DXDXE^318^. Moreover, the molecular docking model of *Ma*Chi1 and (GlcNAc)_6_ further indicated that Glu^318^ participated in the catalytic process in the catalytic center. The CBM2 domain in *Ma*Chi1 enhanced the binding of the enzyme to chitin, which in turn facilitated the enzymic hydrolysis of the substrate. In addition to chitinous substrates, chitosan, cellulose, and soluble starch are also degraded by *Ma*Chi1. The good thermal stability and wide pH tolerance range exhibited by *Ma*Chi1 make it more advantageous for use in biocatalytic processes. When CC was used as a substrate, *Ma*Chi1 cleaved the glycosidic bonds in a non-processive manner, yielding high levels of GlcNAc and (GlcNAc)_2_. After 12 h of processing, the conversion of CC reached 75.9%, with the yields of GlcNAc and (GlcNAc)_2_ being 227.2 and 505.9 mg/g chitin, respectively. Overall, the excellent properties of *Ma*Chi1 highlight its potential application in biocatalytic processes.

## Figures and Tables

**Table 2:** Substrate spectrum of *Ma*Chi1.

Substrate	Specific Activity (U/mg)
*α*-Chitin	0.50 ± 0.01
*β*-Chitin	1.44 ± 0.02
Colloidal chitin	1.78 ± 0.03
Shrimp shell powder	0.69 ± 0.00
(GlcNAc)_2_	ND ^a^
(GlcNAc)_3_	1.15 ± 0.03
(GlcNAc)_4_	1.28 ± 0.02
(GlcNAc)_5_	1.85 ± 0.01
(GlcNAc)_6_	3.11 ± 0.05
Chitosan (DD 95%)	0.19 ± 0.01
Chitosan (DD 90%)	0.12 ± 0.01
Chitosan (DD 85%)	0.25 ± 0.01
Chitosan (DD 80%)	0.39 ± 0.01
Chitosan (DD 70%)	0.69 ± 0.01
Chitosan (DD 55%)	1.38 ± 0.02
Cellulose	0.34 ± 0.02
Soluble starch	0.34 ± 0.00

^a^ ND, no enzymatic activity was detected.

**Table 3:** Kinetic parameters of *Ma*Chi1 toward colloidal chitin.

Substrate	*V_max_* (μmol·min^−1^·mg^−1^)	*K_m_* (mg/mL)	*K_cat_* (s^−1^)	*K_cat_/K_m_* (mg^−1^·mL·s^−1^)
Colloidal chitin	2.33	4.60	0.43	0.09

## Data Availability

All data are contained within this article; further inquiries can be directed to the corresponding author.

## Supplementary Materials

The following supporting information can be downloaded at https://www.mdpi.com/article/10.3390/md22060287/s1, Figure S1: PCR products of the *Ma*Chi1 gene from *Micromonospora aurantiaca*; Figure S2: Primary structure of the *Ma*Chi1 protein; Figure S3: Model confidence analysis of *Ma*Chi1; Figure S4: The predicted three-dimensional configuration of *Ma*Chi1; Figure S5: Two-dimensional schematic representation of the interaction model between the (GlcNAc)_6_ molecule and the surrounding residues; Figure S6: SDS-PAGE analysis of the purified *Ma*Chi1; Table S1: Purification summary of recombinant *Ma*Chi1 from *M. aurantiaca*; Table S2: Effect of different metal ions on *Ma*Chi1; Table S3: Effect of chemical agents on *Ma*Chi1; Table S4: The online tools used in this study.
