# Supplementary material for: Heterologous Expression and Characterization of a pH-Stable Chitinase from Micromonospora aurantiaca with a Potential Application in Chitin Degradation
Source: Mar Drugs. 2024 Jun 20;22(6):287. doi: 10.3390/md22060287 (PMC11204758; doi:10.3390/md22060287)
Supplement: Supplementary file 1 [file marinedrugs-22-00287-s001.zip › marinedrugs-3052205-supplementary.pdf]

Heterologous Expression and Characterization of a pH-Stable Chitinase from *Micromonospora aurantiaca* with a Potential Application in Chitin Degradation

Han-Zhong Guo [1], Dou Wang [1], Hui-Ting Yang [1], Yu-Le Wu [1], Yong-Cheng Li [1], Guang-Hua Xia [1,2] and Xue-Ying Zhang [1,2]*

[1] Hainan Engineering Research Center of Aquatic Resources Efficient Utilization in South China Sea, Key Laboratory of Food Nutrition and Functional Food of Hainan Province, Key Laboratory of Seafood Processing of Haikou, National R&D Branch Center for Prawn processing technology (Haikou), College of Food Science and Technology, Hainan University, Haikou 570228, China; guohanzhong0622@163.com (H.Z. G.); wangdouu@126.com (D. W.); yanghuiting000701@163.com (H.T. Y.); 17370514528@163.com (Y.L. W.); lyc2360@sina.com (Y.C. L.); xiaguanghua2011@126.com (G.H. X.)

[2] Collaborative Innovation Center of Provincial and Ministerial Co-Construction for Marine Food Deep Processing, Dalian Polytechnic University, Dalian 116034, China

* Correspondence: 994257@hainanu.edu.cn

Contents

Figure S1. PCR products of the *Ma*Chi1 gene from *Micromonospora aurantiaca* 3

Figure S2. Primary structure of the *Ma*Chi1 protein...3

Figure S3. Model confidence analysis of *Ma*Chi1...4

Figure S4. The predicted three-dimensional configuration of *Ma*Chi1.......................4

Figure S5. Two-dimensional schematic representation of the interaction model between the $(GlcNAc)_6$ molecule and the surrounding residues.................................5

Figure S6. SDS-PAGE analysis of the purified *Ma*Chi1 6

Table S1. Purification summary of recombinant *Ma*Chi1 from *M. aurantiaca* 7

Table S2. Effect of different metal ions on *Ma*Chi1 7

Table S3. Effect of chemical agents on *Ma*Chi1...7

Table S4. The online tools used in this study 8

References 9

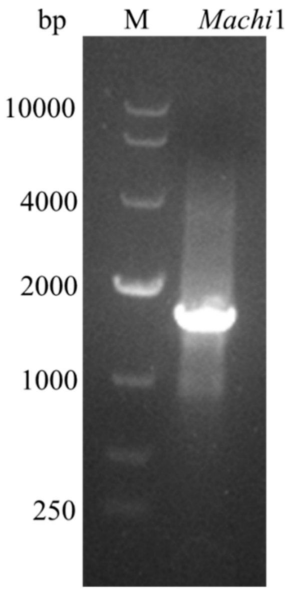

Figure S1. PCR products of *Ma*Chi1 gene from *Micromonospora aurantiaca*. Lane M: DNA size markers; Lane *Machi1*: PCR products of *Ma*Chi1 gene.

```
MKRSLRRALWATGAVVALAVAAVPMTTASAAGSVTATFAKVQDWGTGHEGRVTVTNGTSS  60
TLNGWRIEFTLPSGTSISSSWDADITRSGDRYVAVNKSWASTLAPGASFSWGYNGTGAYK  120
APVSCTINGGSCTGGGTPTTTAPTTAPPTTTPPTTTPPTTTPPTTQPPTTPPPGGKKIVG  180
YFAEWGVYARNYHVKNIHTSGSAAKLTHILYAFGNTTGGRCSIGDSYADYEKAYTAADSV  240
DGVADTWDQPLRGSFNQLRKLKKMYPNLKVLWSFGGWTWSGGFTQAAQNPAAFADSCYSL  300
VKDPRWADVFDGIDIDWEYPNACGLQCDSSGPNAFKNVISALRSKFGSSFLVTAAITADG  360
SNGGKIDATDYAGAANNLNWLMPMTYDYFGAFNAQGPTAPHSPLYSYAGIPQQGFWSDAA  420
IQKLKSKGVPSSKLLLGVGFYGRGWTGVTQAAPGGSATGAAPGTYEAGIEDYKVLKNSCP  480
ATGTVGGTAYAKCGSNWWSYDTPSTIGGKMSYANSQGLGGAFFWELSGDTSNGELIGAIK  540
GGLG  544
```

Figure S2. Primary structure of the *Ma*Chi1 protein. The amino acid sequence of *Ma*Chi1 is shown, highlighting the following regions: N-terminal signal peptide (in green), cellulose binding domain (in yellow), Thr/Pro-rich linker (in gray), GH18 domain (in blue), and C-terminal extension (in black). Conserved tryptophan residues in the cellulose binding domain that are involved in substrate binding are marked with (·) sign, whereas the structural motif in the GH18 domain that are involved in catalysis is boxed by a black line. The number of the last residue in each row is shown on the right side of the sequence.

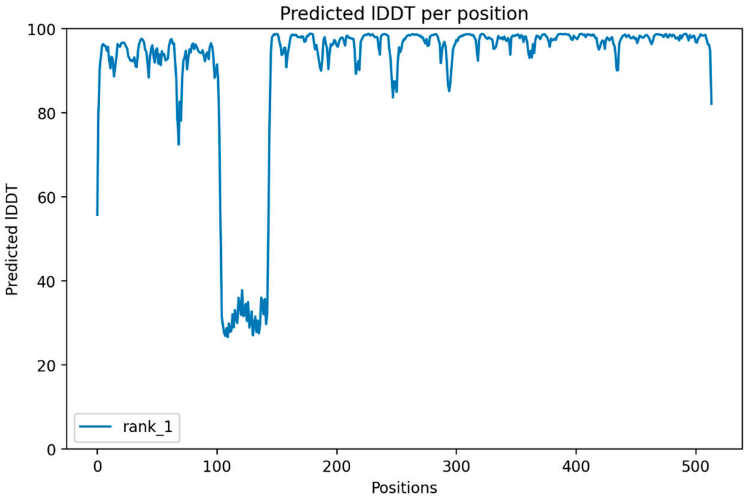

Figure S3. Model confidence analysis of *Ma*Chi1. In the model produced by AlphaFold, per-residue confidence metric is defined as predicted local distance difference test (pLDDT) on a scale from 0 to 100. On this basis, pLDDT score higher than 90 is taken as the high accuracy, pLDDT score between 70 and 90 corresponds to a generally correct backbone prediction, and pLDDT score lower than 70 is considered as low-confidence region. In the AlphaFold models, the linkers that responsible for connecting domains are not always predicted at high confidence (pLDDT > 70)[1,2].

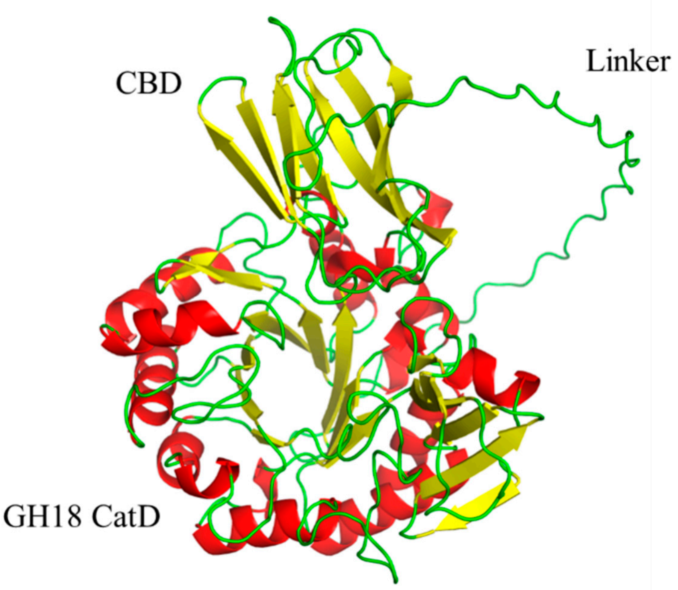

Figure S4. The predicted three-dimensional configuration of *Ma*Chi1. The N-terminal cellulose binding domain (CBD), C-terminal GH18 catalytic domain (GH18 CatD) and linker are labelled. The modeled structure comprised 514 residues, from Ala[31] (at the N-terminal end) to Gly[544] (at the C-terminal end).

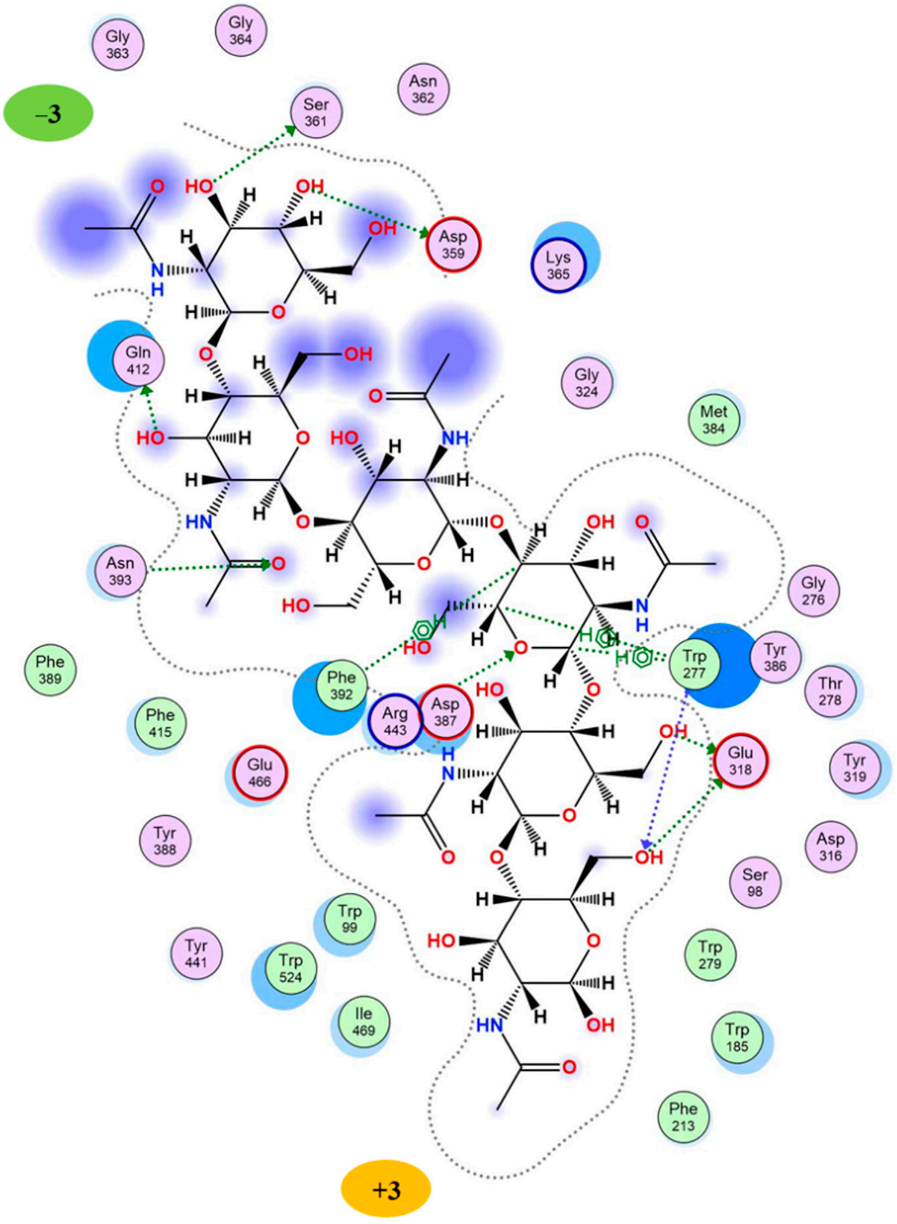

Figure S5. Two-dimensional schematic representation of the interaction model between the $(GlcNAc)_6$ molecule and the surrounding residues. Some residues involved in hydrogen bonds (green or blue dashed line with an arrow) and CH–π interaction (green dashed line) are shown. Monosaccharide units are numbered from +3 (reducing end) to −3 (non-reducing end). The diagram was generated using the program molecular operating environment (MOE).

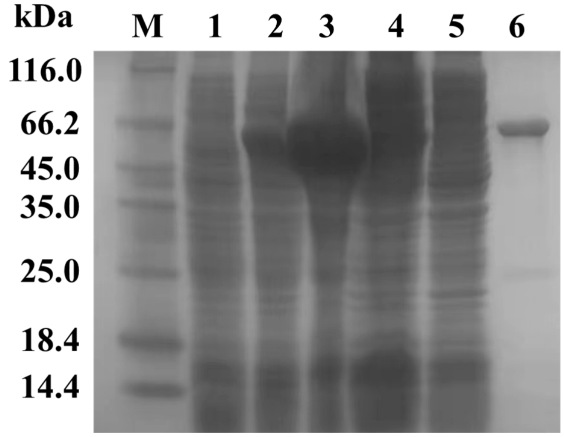

Figure S6. SDS-PAGE analysis of the purified *Ma*Chi1. Lane M, protein standard marker; Lane 1, total protein of *E. coli* BL21(DE3); Lane 2, total protein of recombinant *E. coli* BL21(DE3); Lane 3, lysate supernatant of recombinant *E. coli* BL21(DE3); Lane 4, lysate precipitation of recombinant *E. coli* BL21(DE3); Lane 5, protein eluted with wash buffer from the Ni Sepharose column; Lane 6, purified *Ma*Chi1.

Table S1 Purification summary of recombinant *Ma*Chi1 from *M. aurantiaca*.

Purification step	Total activity (U)	Total protein (mg)	Specific activity (U/mg)	Purification fold	Recovery yield (%)
Crude enzyme	1.3	5.6	0.2	1	100
Ni Sepharose	1.1	0.2	5.5	27.5	84.6

Note: In this experiment, 100 mL of fermentation broth was used.

Table S2 Effect of different metal ions on *Ma*Chi1

Metal ions	Relative activity (%)	
	1 mM	5 mM
Control	100	100
K^+	87.5 ± 1.1	49.1 ± 1.1
Ag^+	46.1 ± 0.4	67.3 ± 2.6
Mg^{2+}	103.4 ± 3.5	113.6 ± 2.7
Fe^{2+}	59.1 ± 2.5	40.8 ± 1.5
Ca^{2+}	108.8 ± 4.1	87.9 ± 2.5
Co^{2+}	85.6 ± 2.8	54.9 ± 2.6
Ba^{2+}	109.0 ± 2.8	92.8 ± 2.6
Zn^{2+}	94.6 ± 2.4	67.9 ± 2.3
Cu^{2+}	46.9 ± 1.1	40.5 ± 0.8
Fe^{3+}	77.3 ± 1.6	35.0 ± 1.6

Table S3 Effect of chemical agents on *Ma*Chi1

Chemical agents	Concentration	Relative activity (%)
Control	0	100
Urea	200 mM	85.8 ± 2.0
β-Mercaptoethanol	1% (v/v)	104.1 ± 3.2
DTT	10 mM	144.8 ± 2.7
EDTA	10 mM	102.6 ± 2.3
SDS	10 mM	61.9 ± 2.8
Tween-20	1% (v/v)	96.5 ± 2.6
Tween-40	1% (v/v)	106.5 ± 2.7
Tween-60	1% (v/v)	74.9 ± 1.6
Tween-80	1% (v/v)	101.0 ± 3.6
Triton X-100	1% (v/v)	107.2 ± 1.1

Table S4 The online tools used in this study

Online tools	Purpose	Website address
The signalP 5.0 server	Examine the signal peptides	https://www.cbs.dtu.dk/services/SignalP/
The CD-Search of NCBI	Analyze the conserved domains	https://www.ncbi.nlm.nih.gov/cdd/
The ExPASy ProtParam program	Predict the theoretical molecular weights and isoelectric points	https://web.expasy.org/protparam/

References

[1] Tunyasuvunakool, K.; Adler, J.; Wu, Z.; Green, T.; Zielinski, M.; Zídek, A.; Bridgland, A.; Cowie, A.; Meyer, C.; Laydon, A.; et al. Highly accurate protein structure prediction for the human proteome. Nature 2021, 596, 590.

[2] David, A.; Islam, S.; Tankhilevich, E.; Sternberg, M. The alphafold database of protein structures: A biologist's guide. J. Mol. Biol. 2022, 434, 167336.
